# A systematic review on the effects of group singing on persistent pain in people with long‐term health conditions

**DOI:** 10.1002/ejp.1485

**Published:** 2019-10-15

**Authors:** J. Yoon Irons, David Sheffield, Freddie Ballington, Donald E. Stewart

**Affiliations:** ^1^ Health and Social Care Research Centre University of Derby Derby UK; ^2^ Queensland Conservatorium Research Centre Griffith University Brisbane Australia; ^3^ University of Derby Online Learning Derby UK; ^4^ University of Derby Derby UK; ^5^ School of Medicine Griffith University Brisbane Australia

## Abstract

**Background and Objectives:**

Singing can have a range of health benefits; this paper reviews the evidence of the effects of group singing for chronic pain in people with long‐term health conditions.

**Database and Data Treatment:**

We searched for published peer‐reviewed singing studies reporting pain measures (intensity, interference and depression) using major electronic databases (last search date 31 July 2018). After screening 123 full texts, 13 studies met the inclusion criteria: five randomized controlled trials (RCTs), seven non‐RCTs and one qualitative study. Included studies were appraised using Downs and Black and the Critical Appraisals Skills Programme quality assessments.

**Results:**

Included studies reported differences in the type of singing intervention, long‐term condition and pain measures. Due to the high heterogeneity, we conducted a narrative review. Singing interventions were found to reduce pain intensity in most studies, but there was more equivocal support for reducing pain interference and depression. Additionally, qualitative data synthesis identified three key linked and complementary themes: physical, psychological and social benefits.

**Conclusion:**

Group singing appears to have the potential to reduce pain intensity, pain interference and depression; however, we conclude that there is only partial support for singing on some pain outcomes based on the limited available evidence of varied quality. Given the positive findings of qualitative studies, this review recommends that practitioners are encouraged to continue this work. More studies of better quality are needed. Future studies should adopt more robust methodology and report their singing intervention in details. Group singing may be an effective and safe approach for reducing persistent pain and depression in people with long‐term health conditions.

**Significance:**

This systematic review assesses research evidence for the effectiveness of group singing on chronic pain in people with long‐term health conditions. Narrative syntheses revealed that there is partial support for singing effects on some pain outcomes based on the limited available evidence of varied quality. Qualitative data provided additional support of physical, psychological and social benefits. The review highlights implications for practice and future studies.

## 1 INTRODUCTION

Singing requires the active involvement of both vocal apparatus and the respiratory system (Irons, Kenny, McElrea, & Chang, 2012; Irons, Kuipers, & Petoz, 2013; Irons, Petocz, Kenny, & Chang, 2019). Singing encourages the active use of diaphragmatic breathing, which promotes deep and slow breathing. This diaphragmatic breathing influences a number of important physiological functions, such as the cardiovascular system and the autonomic nervous system (Russo, Santarelli, & O’Rourke, 2017). Singing also stimulates both the auditory and sensory‐motor pathways in the brain (Wan, Ruber, Hohmann, & Schlaug, 2010). Recently, increasing number of studies have shown the potential health benefits of group singing programmes for people with long‐term health conditions, such as chronic obstructive pulmonary disease (Bonilha, Onofre, Vieira, Almeida Prado, & Martinez, 2009; Lord et al., 2010, 2012; Morrison et al., 2013; Skingley, Clift, Hurley, Price, & Stephens, 2018) and Parkinson's (Fogg‐Rogers et al., 2016; Stegemoller, Radig, Hibbing, Wingate, & Sapienza, 2017). However, the role of singing in chronic health conditions with persistent pain is unclear.

Chronic pain is defined by its persisting for more than 3 months in one or more parts of the body (Treede et al., 2015). Chronic pain is a multi‐dimensional experience, involving not only biological but also sensory, cognitive and affective processes, which negatively affects one's physical, emotional and social functioning (Gatchel, Peng, Peters, Fuchs, & Turk, 2007; Moseley & Butler, 2015). A large European survey study found that chronic pain is prevalent in adults: 19% of survey respondents reported to have moderate to severe pain intensity, and to have changed or lost jobs as well as being diagnosed with depression due to pain (Breivik, Collett, Ventafridda, Cohen, & Gallacher, 2006). Persistent pain due to long‐term health conditions (e.g. Parkinson's, stroke and dementia) is common (Achterberg et al., 2013; Hénon, 2006; Skogar & Lokk, 2016). People living with persistent pain also experience depression, anxiety, anger, reduced self‐efficacy and self‐esteem (Burke, Mathias, & Denson, 2015). Pain is usually recognized as multi‐dimensional comprising at least sensory and affective components as well as having consequences on everyday activities (Melzack & Casey, 1968). One approach to understand the ways in which singing might influence individuals is through Price's Pain Processing model (Price, 1999; Riley et al., 2002). This model consists of a number of stages: first, an initial sensory‐discriminative stage, where the major component is the perceived intensity of the pain sensation; second, a stage of pain processing, unpleasantness, reflects an individual's immediate affective response and involves limited cognitive processing; third, there is a stage of pain processing that involves longer term reflective or cognitive processes that relate to the meanings or implications that pain holds for one's life (Price, 1999) as manifested by negative emotions related to pain (e.g. depression and anxiety). The final component of this model concerns overt behavioural expressions of pain (e.g. ability to participate in daily responsibilities), which accord with quality of life measures, particularly when they pertain to activities of daily life and interference. Focusing on these different components may aid understanding of how singing influences pain processing.

It is plausible that singing will influence pain as singing increases body relaxation and reduces stress levels (Ma et al., 2017). A recent study found that people who practiced relaxing deep slow breathing patterns had increased pain thresholds and reduced pain sensitivity (Busch et al., 2012). Singing, compared with passively listening to music, also activates the body's own pain relief function (endorphins) and elevates positive mood (Dunbar, Kaskatis, MacDonald, & Barra, 2012). Further, singing might influence pain processing through distraction—focusing attention away from pain may reduce the impact of pain and how it interferes with activities of daily living (Blomqvist & Hallberg, 2002). Finally, singing is a group activity that leads to social cohesion and emotional support (Pearce et al., 2016; Tarr, Launay, & Dunbar, 2014; Weinstein, Launay, Pearce, Dunbar, & Stewart, 2016), which has been related to pain (Bernardes, Forgeron, Fournier, & Reszel, 2017; Brown, Sheffield, Leary, & Robinson, 2003; Pearce et al., 2016; Tarr et al., 2014; Weinstein et al., 2016). Using the pain processing model domains, this review aims to answer: To what extent does singing influence pain intensity, unpleasantness, interference and depressive symptoms in people with long‐term health conditions?

## 2 METHODS

### 2.1 Inclusion and exclusion criteria

Given singing interventions are relatively new in the field of pain, we aimed to quantify the field and identify the research gaps. Hence, we included non‐controlled studies as well as randomized controlled trials (RCTs). We included peer‐reviewed singing studies with people with long‐term health conditions associated with persistent pain: for example, arthritis, cardiovascular diseases, cancers, chronic respiratory diseases, diabetes, fibromyalgia and dementia (Wimo, Jönsson, Bond, Prince, & Winblad, 2013). We also included studies involving cancer patients, as cancer is associated with persistent pain. We excluded long‐term mental health conditions, as a recent systematic review on the effects of singing for mental health service users has been published (Williams, Dingle, & Clift, 2018). We excluded studies of professional singers and studies with children and adolescent samples. We also excluded studies that had very brief singing interventions (<2 weeks), had interventions that were not facilitated by professionals with a relevant qualification (e.g. music therapist, professional singing teachers, speech therapists, musicians, nurses or occupational therapists) or did not collect pre‐ and post‐intervention pain data. We did not have language restrictions.

### 2.2 Types of outcome measures

Primary outcomes: Pain intensity, unpleasantness, pain interference and depression associated with pain measured by validated questionnaires, such as the Brief Pain Inventory (BPI) and Hospital Anxiety Depression Scale (HADS).

Based on the framework of Price's Pain Model (Price, 1999; Riley et al., 2002) and consistent with current research in chronic pain (Aaron, Fisher, de la Vega, Lumley, & Palermo, 2019), we chose these pain outcomes. Pain intensity is “magnitude of experienced pain” (Cook et al., 2013); unpleasantness is individual's immediate affective response (e.g. how unpleasant/horrible the pain is) (Riley et al., 2002). Pain interference refers to functional limitations, i.e., how much does pain impact on physical function, work, recreation, social activities, family roles, activities of daily living and sleep (Cook et al., 2013). Depression commonly coexists with chronic pain; can further aggravate the severity of pain experience as well as depression (Sheng, Liu, Wang, Cui, & Zhang, 2017). Thus, it is vital to assess depression when assessing pain (Dansie & Turk, 2013).

Secondary outcomes: number of general practitioner (GP)/pain specialist's visits. These were assessed by self‐report.

### 2.3 Search methods

The protocol for this review was registered with the International Prospective Register of Systematic Reviews (PROSPERO) (ID 2016 CRD42016047557). This review was conducted in accordance with the Cochrane Systematic Review methods (Higgins & Green, 2011); it is reported using the Preferred Reporting Items for Systematic Reviews and Meta‐Analyses (PRISMA) guidelines (Moher, Liberati, Tetzlaff, & Altman, 2009) and in accordance with A MeaSurement Tool to Assess systematic Reviews criteria (Shea et al., 2007), to allow for evaluation and to reduce the potential for bias in the review.

Major database searches were performed by the authors with experience of conducting systematic reviews (JYI, DS and FB), using key words, based on advice from experienced librarians, related to singing, chronic health conditions and pain (Please see Appendix S1 for the detailed search terms). Databases searched included Cochrane Database of Systematic Reviews, Cochrane Central Register of controlled trials, MEDLINE (EBSCO platform), PUBMED, CINAHL, WEB OF SCIENCE, PSCYINFO, SCOPUS, CORE* and Google Scholar* (*first 20 most relevant studies were inspected). In addition, we searched clinical trials registry (http://www.clinicaltrials.gov/), Dimensions (http://www.dimensions.ai/) and Repository for Arts and Health Resources (http://www.artshealthresources.org.uk/) for relevant studies. We inspected the reference lists of relevant studies and citing articles to capture any articles missed through our database searches. The end date for searches was 31 July 2018.

### 2.4 Data collection and analysis

Two review authors (JYI & DS) screened the eligibility of studies for inclusion in the review, based on the inclusion criteria. Any disagreements were resolved in consultation with a third author (FB); however, there were no discrepancies.

Two review authors (JYI & DS) independently extracted data from a data collection form, including citation details, trial setting, inclusion and exclusion criteria, participants (e.g. age, gender, health condition, related pain history), intervention details (e.g. intensity and duration of intervention, types of singing, lists of songs, information on facilitators, etc.), outcome measures (e.g. self‐reported pain measures, depression) and results (statistical techniques used, p values and effect sizes). We contacted study authors for any missing data and further information. A third author (FB) independently reviewed the extracted data to ensure accuracy and reliability, with reviewers meeting to confirm agreement of extraction and to establish reliability. Where there were discrepancies, these were resolved by discussion; agreement was high (Kappa = 0.98).

Methodological quality of the included trials was assessed by two independent reviewers (JYI & FB) and a third author was consulted when there were discrepancies. For both RCTs and non‐RCTs, we have utilized a modified Downs and Black quality assessment checklist to assess study qualities (Downs & Black, 1998). We simplified the question No.27 where we scored one for carrying out a power calculation and zero for no power calculation based on previous recommendations (Kennelly, 2011) (Please see Appendix S2). Further, a Critical Appraisals Skills Programme (CASP) checklist was employed to assess qualities in the studies which reported qualitative data (CASP, 2018). We graded studies' qualities as poor, fair and good according to their scores of either the Downs and Black or CASP checklist.

### 2.5 Data synthesis

Effect sizes were extracted for each study and, where necessary, were calculated using means, standard deviations and sample sizes at baseline and post‐intervention of experimental and control conditions (Decoster & Claypool, 2004). Where such statistics were missing, we used F‐statistics, *t*‐values and *p*‐values to calculate effect sizes; we used Cohen’s (1988) suggestions to categorize effect size as small, medium and large. RCT effects were categorized using “significantly favours intervention,” “trends towards intervention,” “no difference,” “trends towards control” and “significantly favours control” (Cooper, Hedges, & Valentine, 2009) Authors were contacted when relevant data were not reported in the article; the following authors provided additional data: Kenny and Faunce (2004); Clements‐Cortes (2013, 2015); Grape, Töres, Britt‐Maj, and Rolf (2009); Morrison et al. (2013); Pongan et al. (2017); Tamplin (2013); Stegemoller (2017) and Reagon (2017). We conducted narrative syntheses based on the framework by Popay and colleagues (Popay et al., 2006).

## 3 RESULTS

### 3.1 Description of studies and results of searches

**Figure 1:**
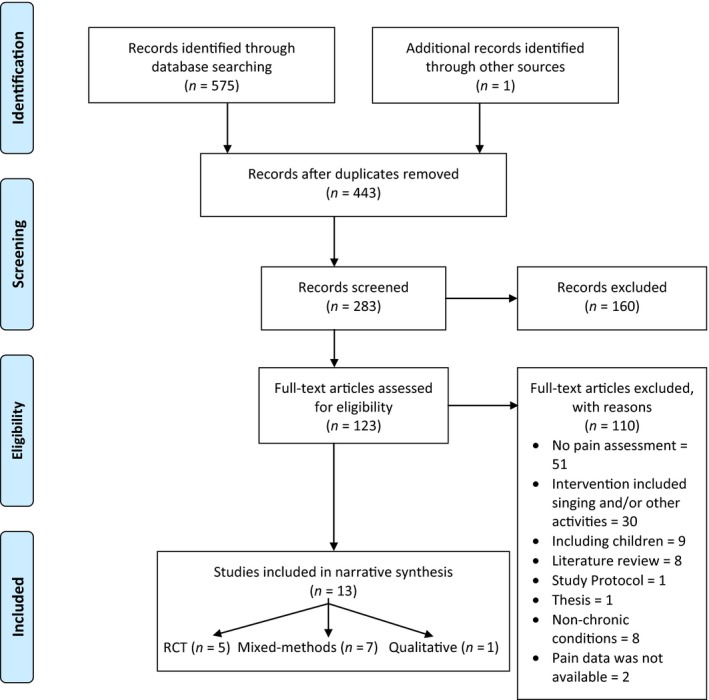
PRISMA flow diagram

The electronic database searches found 575 records. After screening abstracts and full‐text review, 13 studies met the inclusion criteria (Figure 1 for PRISMA flow diagram) including five randomized controlled trials (RCTs) (Bradt, Norris, Shim, Gracely, & Gerrity, 2016; Grape et al., 2009; Kenny & Faunce, 2004; Pongan et al., 2017; Tamplin et al., 2013); seven non‐randomized controlled trials (non‐RCTs) (Clements‐Cortes, 2013, 2015; Fogg‐Rogers et al., 2016; Gale, Enright, Reagon, Lewis, & Van Deursen, 2012; Morrison et al., 2013; Reagon et al., 2017; Stegemoller et al., 2017) and one qualitative study (Hopper, Curtis, Hodge, & Simm, 2016). All RCTs and non‐RCTs reported the effects of group singing on pain intensity and/or interference. Two studies recruited chronic pain patients (Bradt et al., 2016; Kenny & Faunce, 2004), three studies involved old people with Alzheimer's disease (Clements‐Cortes, 2013, 2015; Pongan et al., 2017), two studies recruited cancer survivors (Gale et al., 2012; Reagon et al., 2017) and two studies were with people with Parkinson's and stroke survivors (Fogg‐Rogers et al., 2016; Stegemoller et al., 2017). Descriptions of included studies are presented in Table 1. Two Chronic Obstructive Pulmonary Disease (COPD) trials could not be included in the meta‐analysis, as the trials' authors were unable to provide data on pain interference (SF‐12) (Lord et al., 2010, 2012). One study (Hopper et al., 2016) reported only qualitative findings of group singing interventions for chronic pain patients, while seven studies reported qualitative data within a mixed‐methods design (Bradt et al., 2016; Clements‐Cortes, 2013, 2015; Fogg‐Rogers et al., 2016; Gale et al., 2012; Reagon et al., 2017; Tamplin et al., 2013).

**Table 1:** Description of included studies (*k* = 13)

Study ID (1st author, year, Country)	Diagnosis, number of Participants at baseline, Mean age	Study design, control group condition	Singing intervention frequency and intensity, total hours of singing	Setting, facilitator, repertoires	Pain outcome measures, Pain questionnaire	Depression measure/Questionnaire	Comments
Kenny & Faunce (2004)^a^, Australia	Chronic Pain, *N* = 77, 40.02 years	RCT, Exercises group	0.5‐hr session, three times per week for 3 weeks, 4.5 hr	Chronic Pain Management Centre (Royal North Shore Hospital, Sydney), Singing/piano teacher, Songs with repetitive phrases	Pain interference, (pain disability questionnaire)	Zung depression inventory	
Grape et al. (2009)^a^, Sweden	Irritable Bowel Syndrome, *N* = 55, NR	RCT, Education group	1‐hr session, once weekly for 1 year (46 hr)	Not reported, Not reported, Not reported	Pain Interference (GSRS‐IBS’s Pain subscale)	None	
Gale et al. (2012)^a^, UK	Cancer, *N* = 32, 59 years	Non‐RCT, No comparison group	2‐hr session, once weekly for 12 weeks (24 hr)	Community, Professional musicians, Arranged music	Pain intensity & Pain interference (SF−36 Bodily pain subscale)	Hospital Anxiety Depression Scale (HADS)	Qualitative analysis was performed on interviews.
Tamplin et al. (2013)^a^, Australia	Spinal cord injury, *N* = 24, 45 years	RCT, Music appreciation & relaxation group	1‐hr session, three times per week for 12 weeks (36 hr)	Not reported, Music therapist, Not reported	Pain Intensity (AQoL−4D)	Profile of mood States (POMS)	Qualitative analysis was performed on post‐intervention interviews.
Morrison et al. (2013)^a^, UK	COPD, *N* = 106, 69.5 years	Non‐RCT, No comparison group	1.5‐hr session, once weekly over 36 weeks (54 hr)	Community, Experienced group singing facilitators, Familiar & new songs	Pain Interference (SF‐12 Bodily pain subscale)	EQ‐5D (depression/anxiety subscale)	Participants' written comments about their experience were presented.
Clements‐Cortes (2013)^a^, Canada	Dementia, *N* = 28, 72.9 years	Non‐RCT, No comparison group	1‐hr session, once weekly for 16 weeks (16 hr)	Adult day‐care centre, Music therapist, Familiar & simple songs that participants chosen	Pain Intensity & Pain Interference (AQoL)	None	Qualitative analysis was performed on interview transcripts.
Clements‐Cortes (2015)^a^, Canada	mild to moderate Alzheimer's Disease, *N* = 35, range 66–99 years	Non‐RCT, (Group 1) older people with AD; (Group 2) significant others/staff/volunteers	1‐hr session, once weekly for 16 weeks (16 hr)	Long‐term care facility, Music therapist, Participants' preferred songs from the 1930s to 1940s	Pain Intensity Scale (adopted from FLACC Scale)	None	Qualitative analysis was performed on interview transcripts.
Bradt et al. (2016), USA	Chronic Pain, *N* = 55, 53.75 years	RCT, Wait‐list group	1‐hr session, once weekly for 8 weeks (8 hr)	Health Centre, Music therapist, Inspirational songs chosen by participants; Circle songs; Vocal improvisation	Pain Interference (Westhaven‐Yale Multidimensional Pain Inventory)	Hospital Anxiety Depression Scale (HADS)	Qualitative analysis was performed on focus group transcripts.
Fogg‐Rogers et al. (2016)^a^, New Zealand	Parkinson's & Stroke, *N* = 23 (*N* = 9 carers), 64.4 years	Non‐RCT, No comparison group	1 hr, once weekly for 12 weeks (12 hr)	Community, Music therapist, Not reported	Pain Interference (WHO‐QOL_Bref)	None	Qualitative analysis was performed on interview transcripts.
Hopper et al. (2016), UK	Chronic pain, *N* = 7, range 44–79 years	Non‐RCT, Qualitative study	Weekly ongoing choir	Community Pain Service Centre, A service‐user (person with chronic pain), Not reported	None	None	Qualitative analysis was performed on interview transcripts.
Pongan et al. (2017)^a^, France	Alzheimer's Disease, *N* = 77, 78.8 years (singing group); 80.2 years (control group)	RCT, Painting group	2‐hr session, once weekly for 12 weeks (24 hr)	Memory clinics located in University Hospitals, Professional choir conductor, Well‐known songs chosen by participants	Pain Intensity (EQ−5D); Pain Interference (BPI)	Geriatric Depression Scale (GDS)	
Stegemoller et al. (2017)^a^, USA	Parkinson's, *N* = 27, 67 years	Non‐RCT, (Group 1) high intensity singing; (Group 2) low intensity singing	(Group 1) = 1 hr, twice weekly for 8 weeks (16 hr), (Group 2) = 1‐hr session, once weekly for 8 weeks (8 hr)	Community, Music therapist, popular songs	Pain Interference (WHO‐QOL_Bref)	None	
Reagon et al. (2017)^a^, UK	Cancer, *N* = 816 (*N* = 222 cancer patients, *N* = 593 carers), 62 years (cancer patients); 56 years (carers)	Non‐RCT, No comparison group	1.5‐hr session, once weekly for 12 weeks (18 hr)	Community, Professional musicians, Contemporary & traditional songs	Pain intensity & Pain interference (SF‐36 Bodily pain subscale)	Hospital Anxiety Depression Scale (HADS)	Qualitative analysis was performed on interviews and focus groups data.

Abbreviations: AQoL, Assessment of Quality of life; BPI, Brief Pain Inventory; COPD, Chronic Obstructive Pulmonary Disease; EQ‐5D, EuroQoL‐5D; FLACC Scale, Face Legs Activity Cry Consolability Scale; GSRS‐IBS, Gastrointestinal Symptom Rating Scale for IBS; hrs, hours; IBS, Irritable Bowel Syndrome; *N*, number; NR, Not Reported; RCT, Randomized Controlled Trial; SF‐12, Short Form Health Survey 12; SF‐36, Short Form Health Survey 36; WHO‐QOL_Bref, World Health Organization Quality of Life Scale; yrs, years.

a 1st or corresponding author was contacted to obtain additional data.

Twelve included studies, with a total of 311 participants, evaluated the effects of group singing on pain intensity and/or pain interference, and seven of those studies also reported the effects of singing on depression; no included study reported pain unpleasantness (Tables 2 and 3). All studies were carried out in developed countries. Singing programmes were facilitated by music therapists, experienced professional musicians or a service‐user, a person with chronic pain plus musical background (Hopper et al., 2016), and provided at community centres or hospital/health centres. Singing intervention length varied between 3 weeks and 1 year. The duration of each session was commonly between 60 and 90 min, although Kenny's programme comprised 30‐min sessions (Kenny & Faunce, 2004). Most interventions were conducted weekly (Bradt et al., 2016; Gale et al., 2012; Grape et al., 2009; Morrison et al., 2013; Pongan et al., 2017; Reagon et al., 2017; Stegemoller et al., 2017); however, one met thrice weekly (Tamplin et al., 2013), while one assembled three or more times weekly (Kenny & Faunce, 2004). The interventions were delivered predominantly by an individual with relevant musical skill, such as music therapists (Bradt et al., 2016; Clements‐Cortes, 2013, 2015; Fogg‐Rogers et al., 2016; Stegemoller et al., 2017; Tamplin et al., 2013) or professional musicians (Gale et al., 2012; Grape et al., 2009; Kenny & Faunce, 2004; Morrison et al., 2013; Pongan et al., 2017; Reagon et al., 2017). The majority of studies provided little details of the singing interventions; two studies provided no information on repertoires (Grape et al., 2009; Hopper et al., 2016) (Table 1).

**Table 2:** Included studies' results on pain intensity, interference and depression measures and quality assessments

Study ID (1st Author, Year, Country)	Pain questionnaire	Pain questions	Effect size (*g*) (95% Confidence Intervals)	Participants Number	Quality Assessment§
Pain intensity					
Tamplin et al. (2013)^a^ Australia	AQoL−4D (Assessment of Quality of Life−4D)	“How much pain or discomfort do I experience?”	−0.18 (−0.75–0.39)	13	23
Morrison et al. (2013) UK	EQ−5D (EuroQual−5D)	“I have no pain/slight pain/moderate pain/severe pain or extreme pain.”	−0.33 (−0.61–0.05)	69	11
Clements‐Cortes (2015) Canada	Author has used a visual analogue scale adopted from FLACC Scale.	Visual Analogue Scale 0 = no pain ‐ 5 = a lot of pain	−0.63* (−1.23–−0.03)	14	11
Pongan et al. (2017)^a^ France	EQ−5D (EuroQual−5D)	“I have no pain/slight pain/moderate pain/severe pain or extreme pain.”	−0.47* (−0.89–−0.04)	25	24
Pain interference					
Kenny and Faunce (2004)^a^ Australia	Pain Disability Questionnaire	“Does your pain interfere with your normal work/personal care/travelling?”	−0.53 (−1.14–0.07)	13	18
Grape et al. (2009)^a^ Sweden	GSRS‐IBS's Pain subscale (Gastrointestinal Symptom Rating Scale modified for use in patients with Irritable Bowel Syndrome)	“Have you been bothered by abdominal pain during past week?”	−0.25 (−0.63–0.13)	28	14
Morrison et al. (2013) UK	SF−12 (Short Form−12)	“During the past 4 weeks, how much did pain interfere with your normal work (including work outside the home and housework?”	−0.19 (−0.41–0.03)	71	11
Fogg‐Rogers et al. (2016) New Zealand	WHOQOL_BREF (World Health Organization‐Quality Of Life‐Bref)	“To what extent do you feel that physical pain prevents you from doing what you need to do?”	0.38 (−0.21–0.96)	13	9
Bradt et al. (2016)^a^ USA	Westhaven‐Yale Multidimensional Pain Inventory	“How much does your pain problem interfere with your day to day activities?”	−0.65* (−1.13–−0.18)	22	19
Pongan et al. (2017)^a^ France	BPI (Brief Pain Inventory)	“How, during the past 24 hr, pain has interfered with your general activity/mood/walking ability/normal work/relationship with others/sleep?”	0.01 (−0.25–0.27)	31	24
Stegemoller et al. (2017) USA	WHOQOL_BREF (World Health Organization‐Quality Of Life‐Bref)	“To what extent do you feel that physical pain prevents you from doing what you need to do?”	−0.38* (−0.76–0.00)	29	12
Pain intensity + interference					
Gale et al. (2012) UK	SF−36 (Short Form−36)	“How much bodily pain have you had during the past 4 weeks?” “During the past 4 weeks, how much did pain interfere with your normal work (including both work outside of the home and housework)?”	−0.36 (−0.83–0.10)	20	11
Clements‐Cortes (2013) Canada	AQoL (Assessment of Quality of Life)	“How much pain or discomfort do you experience?” “How often does pain interfere with your usual activities?”	−0.15 (−0.53–0.23)	28	11
Reagon (2017) UK	SF−36 (Short Form−36)	“How much bodily pain have you had during the past 4 weeks?” “During the past 4 weeks, how much did pain interfere with your normal work (including both work outside of the home and housework)?”	−0.23 (−0.40–0.03)	60	11
Depression					
Kenny and Faunce (2004)^a^ Australia	Zung Depression Inventory	“I feel down‐hearted and blue–a little of the time/some of the time/good part of the time/most of the time.”	−0.61 (−1.25–0.03)	12	18
Gale et al. (2012) UK	HAD (Hospital Anxiety Depression)	“I feel cheerful–not at all/not often/sometimes/most of the time”	−0.22 (−0.68–0.23)	20	11
Tamplin et al. (2013)^a^ Australia	POMS (Profile of Mood States) Depression is one of the six domains.	“Below is a list of words that describe feelings people have. Please circle the number that best describes how you feel right now.” For example, Feeling unhappy/sad/hopeless/satisfied/worthless	−0.26 (−0.84–0.31)	13	23
Morrison et al. (2013) UK	EQ−5D	“I am not/slightly/moderately/severely/extremely anxious or depressed”	−0.28* (−0.52–−0.04)	71	11
Bradt et al. (2016)^a^ USA	HAD (Hospital Anxiety Depression)	“I feel cheerful–not at all/not often/sometimes/most of the time”	−0.06 (−48–0.37)	22	19
Pongan et al. (2017)^a^ France	GDS (Geriatric Depression scale)	“Do you feel happy most of the time? – Yes/No”	0.11 (−0.25–0.46)	31	24
Reagon et al. (2017) UK	HAD (Hospital Anxiety Depression)	“I feel cheerful – not at all/not often/sometimes/most of the time”	−0.11 (−0.36–0.15)	60	11

IBS, Irritable Bowel Syndrome.

a Randomized Controlled Trial, *p < .05.

^§^ Downs & Black quality assessment; possible max. score = 28.

**Table 3:** Effect size of RCTs of pain intensity, interference and depression and quality assessment

Study ID (1st Author, Year)	Number of Participants in each group	Comparison (Control group)	Effect size (*g*) (95% Confidence Intervals)	Comments	Quality Assessment§
Pain intensity					
Tamplin et al. (2013)	SG^a^ = 13	Music relaxation/appreciation group (active comparator)	−0.24 (−0.66–0.17)	Trend towards intervention	23
	CG^b^ = 11				
Pongan et al. (2017)^c^	SG^a^ = 31	Painting (active comparator)	−0.15 (−0.41–0.11)	Trend towards intervention	24
	CG^b^ = 28				
Pain interference					
Kenny and Faunce (2004)	SG^a^ = 12	Exercise while listening to the singing group singing practice (active comparator)	0.24 (−0.04–0.52)	Trend towards control	18
	CG^b^ = 39				
Grape et al. (2009)	SG^a^ = 11	Education group (passive comparator)	−0.55 (−0.97–−0.11)	Significantly favours intervention	14
	CG^b^ = 14				
Bradt et al. (2016)	SG^a^ = 22	Wait‐list (passive comparator)	−0.58 (−0.91–−0.26)	Significantly favours intervention	19
	CG^b^ = 22				
Pongan et al. (2017)^c^	SG^a^ = 31	Painting (active comparator)	0.42 (0.15–0.68)	Significantly favours control	24
	CG^b^ = 28				
Depression					
Kenny and Faunce (2004)	SG^a^ = 12	Exercise while listening to the singing group singing practice (active comparator)	0.09 (−0.18–0.37)	No difference	18
	CG^b^ = 39				
Tamplin et al. (2013)	SG^a^ = 13	Music relaxation/appreciation group (active comparator)	−0.18 (−0.23–0.59)	No difference	23
	CG^b^ =11				
Bradt et al. (2016)	SG^a^ = 22	Wait‐list (passive comparator)	−0.27 (−0.54–0.00)	Trend towards intervention	19
	CG^b^ = 22				
Pongan et al. (2017)^c^	SG^a^ = 31	Painting (active comparator)	0.43 (0.16–0.69)	Significantly favours control	24
	CG^b^ = 28				

Note

SG^a^ = Singing Group; CG^b^ = Control Group ^c^ = authors reported Intention‐To‐Treat analysis.

^§^ Downs & Black quality assessment; possible max. score = 28.

Only one study (Bradt et al., 2016) reported including a predominantly African‐American ethnic population. None of the included studies reported the number of GP/pain specialist's visits nor pain unpleasantness. We summarized each study's details of pain and depression measures in Table 2 along with each study's effect size and confidence intervals.

### 3.2 Quality assessments of included studies

Twelve included studies were quality assessed according to the Downs and Black quality assessment tool (Downs & Black, 1998) (Table 4). Overall, 12 included studies were appraised to have low to high qualities: Fogg‐Rogers et al. (2016) scored lowest nine, while Pongan et al., 2017 scored 24 out of a possible 28. Amongst the RCTs, Pongan et al. (2017) and Tamplin et al. (2013) studies were judged to be of good quality, in part as they attempted to blind the experimental condition (singing) by offering active comparators, namely painting (Pongan et al., 2017) and a music appreciation and relaxation programme (Tamplin et al., 2013); blinding assessors was also achieved in both studies. Three RCTs (Bradt et al., 2016; Grape et al., 2009; Kenny & Faunce, 2004) and seven non‐RCTs were unable to blind participants nor assessors. In terms of comprehensive attempt to measure adverse events and describing principal confounders (Question No 5), none of the included studies reported any details (Table 4).

**Table 4:** Quality Assessment of included quantitative studies (k = 12)

Downs & Black quality assessment	Kenny 2004^a^	Grape 2009^a^	Gale 2012^b^	Morrison 2013^b^	Clement‐Cortes 2013^b^	Tamplin 2013^a^	Clement‐Cortes 2015^b^	Bradt 2016^a^	Fogg‐Rogers 2016^b^	Stegemoeller 2017^b^	Reagon 2017^b^	Pongan 2017^a^
Reporting (0–11^c^)												
Q1. Aims	1	1	1	1	1	1	1	1	0	1	1	1
Q2. Outcomes	1	1	1	1	1	1	1	1	0	1	1	1
Q3. Participants	1	1	1	1	1	1	1	1	1	1	1	1
Q4. Intervention	1	0	1	1	1	1	1	1	1	1	1	1
Q5. Confounders^d^	0	0	0	0	0	0	0	0	0	0	0	0
Q6. Findings	1	1	1	1	1	1	1	1	1	1	1	1
Q7. Variability	1	0	0	0	0	1	0	1	0	0		1
Q8. Adverse event	0	0	0	0	0	0	0	0	0	0	0	0
Q9. Drop‐outs	0	0	1	1	1	1	1	1	1	1	1	1
Q10. Probability	1	1	1	1	1	1	1	1	1	1	1	1
Reporting sub‐scores	7	5	7	7	7	8	7	8	5	7	7	8
External validity (0–3^c^)												
Q11. Participants	1	1	0	0	0	1	0	1	0	0	0	1
Q12. Population	0	0	0	0	0	0	0	0	0	0	0	0
Q13. Treatment	1	0	0	0	0	1	0	1	0	0	0	1
External validity sub‐scores	2	1	0	0	0	2	0	2	0	0	0	2
Internal validity (0–7^c^)												
Q14. intervention	0	0	0	0	0	1	0	0	0	0	0	1
Q15. blinding	0	0	0	0	0	1	0	0	0	0	0	1
Q16. results	1	1	1	1	1	1	1	1	1	1	1	1
Q17. follow‐up	1	1	0	0	0	1	0	1	0	0	0	1
Q18. statistics	1	1	1	1	1	1	1	1	1	1	1	1
Q19. compliance	1	1	1	1	1	1	1	1	1	1	1	1
Q20. measures	1	1	1	1	1	1	1	1	1	1	1	1
Internal validity sub‐scores	5	5	4	4	4	7	4	5	4	4	4	7
Selection Bias (confounding) (0–6^c^)												
Q21. Participants	1	1	0	0	0	1	0	1	0	0	0	1
Q22. Time	1	1	0	0	0	1	0	1	0	0	0	1
Q23. Randomization	1	1	0	0	0	1	0	1	0	0	0	1
Q24. Concealment	0	0	0	0	0	1	0	0	0	0	0	1
Q25. Analysis	0	0	0	0	0	0	0	0	0	1	0	1
Q26. Follow‐up	0	0	0	0	0	1	0	1	0	0	0	1
Selection bias sub‐scores	3	3	0	0	0	5	0	4	0	1	0	6
Power^e^ (0–1^c^)												
Q27. Calculation	1	0	0	0	0	1	0	0	0	0	0	1
Overall (0–28^c^)	**18**	**14**	**11**	**11**	**11**	**23**	**11**	**19**	**9**	**12**	**11**	**24**
Rating	**fair**	**fair**	**fair**	**fair**	**fair**	**good**	**fair**	**fair**	**poor**	**fair**	**fair**	**good**

a Randomized Controlled trial.

b Non‐randomized controlled trial.

c possible total scores within each sub‐domains.

d Yes (2), Partially (1), No (0).

e Scored Yes (1), if the study carried out a power calculation; scored No (0), if the study carried out no power calculation.

In addition, we assessed studies reporting qualitative data using CASP qualitative checklist (CASP Programme, 2018). Seven studies (Bradt et al., 2016; Clements‐Cortes, 2013, 2015; Fogg‐Rogers et al., 2016; Gale et al., 2012; Reagon et al., 2017; Tamplin et al., 2013) reported qualitative data as part of a mixed‐methods study design, while Hopper et al. (2016) was the only qualitative study (Table 5). We appraised qualitative studies, with the exception of Fogg‐Rogers et al. (2016), as having good qualities: studies included clear statements of the aims, appropriate methodologies and recruitment strategies. Clements‐Cortes (2015) and Hopper et al. (2016) considered possible implications of the relationship between researcher and participants within their study settings; other articles did not address this issue. All eight studies reporting qualitative data employed recognized analysis methods, such as thematic analysis, or interpretative phenomenological analysis. All authors listed representative quotes with the exception of Tamplin (2013) (for further details, please see Appendix S3). All qualitative studies were appraised as making a valuable contribution to the existing knowledge.

**Table 5:** Quality assessment of qualitative studies (using CASP) (*k* = 8)

CASP* Qualitative Checklist	Gale et al. (2012)^a^	Tamplin (2013)^a^	Clements‐Cortes (2013)^a^	Clements‐Cortes (2015)^a^	Hopper (2016)	Bradt (2016)^a^	Fogg‐Rogers et al. (2016)^a^	Reagon et al. (2017)^a^
Q1. Was there a clear statement of the aims of the research?	Yes	Yes	Yes	Yes	Yes	Yes	Yes	Yes
Q2. Is a qualitative methodology appropriate?	Yes	Yes	Yes	Yes	Yes	Yes	Yes	Yes
Q3. Was the research design appropriate to address the aims of the research?	Yes	Yes	Yes	Yes	Yes	Yes	Yes	Yes
Q4. Was the recruitment strategy appropriate to the aims of the research?	Yes	Yes	Yes	Yes	Yes	Yes	Yes	Yes
Q5. Was the data collected in a way that addressed the research issue?	Yes	Yes	Yes	Yes	Yes	Yes	Yes	Yes
Q6. Has the relationship between researcher and participants been adequately considered?	Can't tell	Can't tell	Can't tell	Yes	Yes	Can't tell	Can't tell	Can't tell
Q7. Have ethical issues been taken into consideration?	Yes	Yes	Yes	Yes	Yes	Yes	Yes	Yes
Q8. Was the data analysis sufficiently rigorous?	Yes	Yes	Yes	Yes	Yes	Yes	Yes	Yes
Q9. Is there a clear statement of findings?	Yes	Yes	Yes	Yes	Yes	Yes	No	Yes
Q10. How valuable is the research?	Yes	Yes	Yes	Yes	Yes	Yes	Yes	Yes

Note

CASP*, Critical Appraisals Skills Programme (downloaded from ://casp-uk.net/wp-content/uploads/2018/03/CASP-Qualitative-Checklist-2018_fillable_form.pdf.) Possible answers are Yes/Can't Tell/No.

a Qualitative data were part of a quantitative study.

### 3.3 Relationship between singing and chronic pain

Overall, all included 12 studies have a great heterogeneity in populations, outcome assessment tools, singing interventions (settings, facilitators, repertoires, length and frequencies of sessions) and study designs. Only five included RCTs were assessed to have low to good quality. Inspection of RCTs revealed differences in chronic condition, intervention length and comparator. Due to these differences, it was not possible to conduct a meta‐analysis. Instead, we present a mixed‐methods review, synthesizing current evidence in the effects of singing for persistent pain in people with chronic health conditions (Popay et al., 2006). Tables 2 and 3 present the effect sizes and 95% Confidence Intervals of singing interventions in each study.

### 3.4 Pain intensity

Four studies (Clements‐Cortes, 2015; Morrison et al., 2013; Pongan et al., 2017; Tamplin et al., 2013) involving 121 participants, measured pain intensity before and after group singing interventions. Pain intensity questions include “How much pain or discomfort do you experience? None at all/I have moderate pain/I suffer from severe pain/I suffer unbearable pain” (AQoL‐4D). All four studies found reductions in pain intensity from pre‐ to post‐singing intervention (Clements‐Cortes, 2015; Morrison et al., 2013; Pongan, 2017; Tamplin, 2013). The effect size in studies with people with COPD (Morrison et al., 2013) and dementia (Pongan et al., 2017) was medium, although Morrison et al. (2013) did not have a comparison group and was appraised to be of fair quality. Tamplin's study (2013) of spinal cord injury patients demonstrated a small effect size, whereas Clements‐Cortes (2015) study with Alzheimer's disease showed a large effect size. However, Clements‐Cortes (2015) study was uncontrolled and assessed to be a fair quality study. Inspection of two good quality RCTs' effect sizes and 95% Confidence Intervals (Pongan, 2017; Tamplin, 2013) revealed that there is “trend towards intervention” in pain intensity outcome measures with small effect sizes in both studies (Table 3).

### 3.5 Pain interference

The effects of singing on pain interference were examined in seven quantitative studies with 205 participants (Bradt, 2016; Fogg‐Rogers et al., 2016; Grape et al., 2009; Kenny & Faunce, 2004; Morrison et al., 2013; Pongan et al., 2017; Stegemoller, 2017). These studies utilized pain questionnaires, which asked the degrees of pain interference in a numerical scale: for example, “Mark the box beside the number that describes how, during the past 24 hr, pain has interfered with your general activity, normal work, etc.," (BPI). Pain interference decreased from pre‐ to post‐singing intervention in studies with chronic pain patients (Bradt, 2016), people with Parkinson's (Stegemoeller, 2017), people with COPD (Morrison et al., 2013) and people with irritable bowel syndrome (IBS) (Grape et al., 2009); however, the decrease was only significant in Bradt et al. (2016)*.* Four RCTs yield mixed results: Bradt et al. (2016) and Grape et al. (2009) studies, rated as of less than good quality, reported findings that “significantly favours intervention,” whereas Pongan et al. (2017) findings “significantly favours control” and Kenny's (2004) had a “trend towards control.” Pongan's study (2017) was appraised to be of good quality, whereas Kenny's study (2004) had a number of weaknesses, such as a high attrition rate (only 31% of singing group participants completed the 3‐week long singing programme) and not reporting missing data. Both Grape (2009) and Bradt (2016) RCTs had passive comparator groups (education group and wait‐list, respectively), and had medium intervention effects. In contrast, singing did not result in decreased pain interference compared to painting (Pongan, 2017) or exercise and listening to singing (Kenny, 2004); indeed, painting resulted in larger decreases in interference than singing (Tables 2 and 3).

### 3.6 Pain interference and pain intensity

Three studies measured aspects of both pain intensity and interference simultaneously; both the SF‐36 (Gale et al., 2012; Reagon et al., 2017) and AQoL‐8D (Clements‐Cortes, 2013) have a pain subscale, which includes questions on both pain intensity and pain interference. These two questions' responses are scored together as overall pain scores. Thus, we grouped these studies together. Both cancer studies (Gale, 2012; Reagon et al., 2017) demonstrated reduced pain intensity and inference, although these studies had no comparator groups and were appraised to have fair quality. Clement‐Cortes study with people with Alzheimer's disease (2013) also reported a reduction in this combined pain outcome measure (Table 2). No RCTs included this combined pain measures.

### 3.7 Depression

Four RCTs (Bradt, 2016; Kenny & Faunce, 2004; Pongan, 2017; Tamplin et al., 2013) and two non‐RCTs (Morrison et al., 2013; Reagon et al., 2017) with a total of 229 participants evaluated the effects of singing on depression. Inspection of four RCTs revealed mixed results: Pongon study (2017) indicated “significantly favours control,” whereas Kenny and Faunce (2004) and Tamplin et al. (2013) showed “no difference.” Only one RCT (Bradt et al., 2016), rated as of less than good quality, revealed a “trend towards intervention” (Table 2 and 3).

### 3.8 Synthesis I: RCTs

Three of five RCTs employed active comparators: Kenny and Faunce (2004) compared group singing with an exercise and listening to singing condition, while Pongan et al. (2017) had a painting group comparator and Tamplin (2013) had a music appreciation/relaxation comparator group. These three RCTs with the active comparators yield results favouring the comparator conditions. In Kenny and Faunce (2004) and Pongan et al. (2017) studies, the control groups demonstrated improvement in pain interference, while the singing groups did not. Tamplin et al. (2013) also found no difference between singing and control group on depression measure. Two other RCTs with passive control groups, namely an education (Grape et al., 2009) and wait‐list (Bradt et al., 2016), found significant reductions in pain interference and depression (only in Bradt et al., 2016) in the singing group. Attending an education group does not require active engagement, while painting and exercise groups require physical and mental engagement. Positive trends towards group singing are apparent in the passive group; however, the effects of group singing are not greater than, and in some cases is smaller than, those of painting, exercise or music appreciation/relaxation programmes.

### 3.9 Synthesis II: length of singing intervention

The shortest singing intervention length was less than 1 month in Kenny's study (Kenny & Faunce, 2004). Five studies adopted 3‐month intervention (Fogg‐Rogers et al., 2016; Gale et al., 2012; Pongan et al., 2017; Reagon et al., 2017; Tamplin et al., 2013), while two studies were 2‐month long (Bradt et al., 2016; Stegemoller et al., 2017). Morrison et al. (2013) evaluated 9‐month singing programme, and Grape study (2009) lasted for 1 year. These two longer term studies (Grape et al., 2009; Morrison et al., 2013) found reduced pain interference with small effect sizes. In singing studies lasting less than 6 months, there are conflicting results: Some reported a positive trend towards intervention in pain interference (Bradt et al., 2016; Stegemoller et al., 2017), others reported a trend towards control (Pongan et al., 2017) and yet others reported no difference (Gale et al., 2012; Reagon et al., 2017). Kenny's study (2004) RCT of 3 weeks duration indicated a “trend towards control” in pain interference. Although RCTs and non‐RCTs cannot be compared directly, this synthesis suggests that there is preliminary evidence that longer term interventions (>6‐month, weekly singing) can have positive impact on reducing pain intensity, pain interference and depression.

### 3.10 Synthesis III: medical conditions

We also grouped the 12 included quantitative studies according to participants' diagnosis and/or similar conditions. (a) Kenny (2004) and Bradt (2016) recruited patients with a chronic pain diagnosis. (b) Four studies included people with neurological conditions, such as Parkinson's (Stegemoller et al., 2017), Parkinson's and stroke (Fogg‐Rogers et al., 2016) and Alzheimer's disease (Clements‐Cortes, 2013, 2015; Pongan et al., 2017). (c) Two studies included patients with impaired lung function (Morrison et al., 2013; Tamplin et al., 2013). (d) Two cancer studies (Gale et al., 2012; Reagon et al., 2017) and one IBS (Grape et al., 2009) study formed one group. In the chronic pain diagnosis group, there are some differences between the two RCTs (Bradt et al., 2016; Kenny & Faunce, 2004): their findings are inconsistent. While Bradt (2016) found reduction in pain interference, Kenny and Faunce (2004) found an increase. In the second group, the neurological conditions, Stegemoeller's study (2017) demonstrated reduced pain interference with medium effect sizes, whereas Fogg‐Rogers' study (2016) did not. Further, Clement‐Cortes (2013) indicated improvement in pain intensity, and pain intensity combined with interference measures. However, Pongan's et al. (2017) findings showed significantly favoured control condition in pain interference and depression, but there was a trend towards intervention for pain intensity. In the third group of patients with impaired lung function (Morrison et al., 2013; Tamplin et al., 2013), the singing groups demonstrated reduced pain intensity with small to medium effect sizes. Lastly, patients with cancer (Gale et al., 2012; Reagon et al., 2017) or with IBS (Grape et al., 2009) also demonstrated reductions in pain: Reagon et al. (2017) and Gale et al. (2012) studies revealed that singing reduced pain intensity and interference with small to medium effect sizes; Grape's study of IBS patients (2009) also yielded reductions in pain interference with a small effect size. Although RCTs and non‐RCTs cannot be directly compared, this synthesis suggests that there is preliminary evidence that group singing is potentially effective in reducing pain in patients with impaired lung function, IBS and cancer. Inconsistent results were found in patients with neurological conditions (Parkinson's, stroke and Alzheimer's disease) and chronic pain. Moreover, there is no evidence of the effects of singing on pain in other long‐term health conditions.

### 3.11 Synthesis IV: qualitative findings

**Figure 2:**
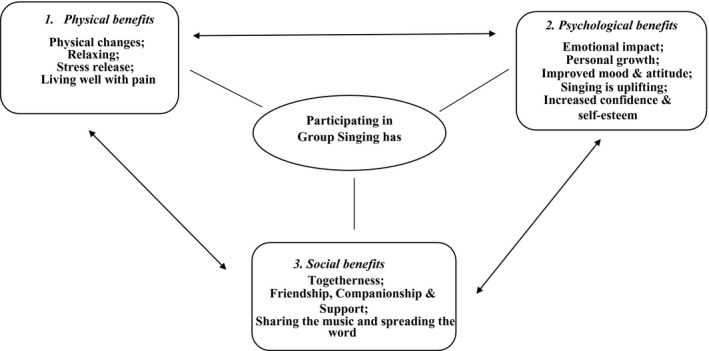
Three key themes from qualitative data

**Figure 3:**
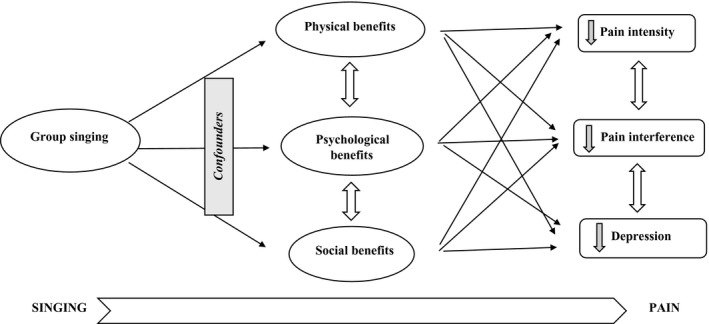
Pathways by which singing improves pain processing

Eight studies explored participants' perspectives of participating in the group singing programme and its impact on their health using a qualitative approach (Bradt et al., 2016; Clements‐Cortes, 2013, 2015; Fogg‐Rogers et al., 2016; Gale et al., 2012; Hopper et al., 2016; Reagon et al., 2017; Tamplin et al., 2013) (see Appendix S3). Hopper et al. (2016) was the only purely qualitative study; the others employed qualitative approach in a mixed‐methods research design. The authors argued that qualitative data would complement the quantitative data, as experience of the group singing programme can be individual and often the depth and intensity of such experience cannot be captured appropriately in the quantitative data. Two studies utilized focus group discussions (Bradt et al., 2016; Reagon et al., 2017), while others conducted semi‐structured interviews with participants (Clements‐Cortes, 2013, 2015; Gale et al., 2012; Hopper et al., 2016; Riley et al., 2002; Tamplin et al., 2013). Overall, singing programmes were well‐received by participants. Through synthesizing qualitative data from eight studies, three key themes were identified: (a) physical benefits; (b) psychological benefits; (c) social benefits. Figure 2 illustrates these key themes and the interactions between them. Participants demonstrated that they experienced positive impacts of singing on the physical, psychological and social aspects and these three domains appear to be interwoven, complementary and correspondent. Increasing positive mood may be due to having friendship/companionship and support, which also impact on reducing stress and anxiety, and increasing relaxation as well as coping with pain. Thus, these themes interact with one another and suggest complex pathways linking group singing to pain (Figure 3).

## 4 DISCUSSION

The current narrative review presented new syntheses of research evidence about the effects of group singing on pain intensity, pain interference and depression in people living with long‐term health conditions. Thirteen studies with 318 participants were included in the review: five RCTs, seven non‐RCTs and one qualitative study. Additionally, seven studies (one RCT, six non‐RCTs) collected qualitative data within a mixed‐methods study design. These studies varied in study design, study quality, sample (variety of long‐term health conditions), pain outcomes measured and details of singing interventions (facilitators, settings, intensities and frequencies of sessions). Due to the high heterogeneity, conducting a meta‐analysis was not appropriate. Therefore, we presented effect size, quality assessments alongside a narrative review. Based on the limited available evidence of varied quality, we conclude there is partial support for singing on some pain outcomes. Specifically, we found in all the studies that there is a positive trend of singing interventions decreasing pain intensity, while there is more equivocal support for singing influencing pain interference and depression.

Analyses of a small subset of five RCTs yielded more mixed effects of singing on pain interference and depression measures. Studies with passive comparator groups (Bradt et al., 2016; Grape et al., 2009) found reductions in pain interference and depression, suggesting that singing may yield benefits compared to education or waiting for the intervention. Studies with active comparator groups (Kenny & Faunce, 2004; Pongan et al., 2017; Tamplin et al., 2013) demonstrated either no difference between singing and control groups, or results favouring control conditions. However, the comparator groups in these studies involved interventions have known positive effects, e.g., exercise (Kenny & Faunce, 2004, or were creative arts interventions likely to activate similar psycho‐social processes as singing, e.g., painting (Pongan et al., 2017). Moreover, there are number of general processes in both active comparative conditions and art making conditions, such as leadership, a focus on meaningful activity and social support from the group (Archer, Buxton, & Sheffield, 2015; Kaptein, Hughes, Murray, & Smyth, 2018).

Qualitative analysis supported and augmented the quantitative data. The qualitative synthesis identified three key themes: physical, psychological and social benefits. As reflected in the qualitative themes (Appendix S3 and Figure 2), singing interventions may offer unique advantages for people with persistent pain. For example, deep breathing is a central element for singing, which can impact on both physiological (e.g. cardiovascular and autonomic nervous system) and emotional functions (Busch et al., 2012; Ma et al., 2017; Russo et al., 2017). Further, studies found that group singing can reduce pain threshold and pain perception (Dunbar, Baron, et al., 2012; Dunbar, Kaskatis, et al., 2012). Other studies also found that group singing had greater social bonding effects than other social activities such as creative writing or craft activity (Pearce, Launay, & Dunbar, 2015; Pearce, Launay, MacCarron, & Dunbar, 2017). Enhanced social bonding is relevant in the pain context, as there is a positive relationship between social isolation and pain interference (Karayannis, Baumann, Sturgeon, Melloh, & Mackey, 2018). Getting together with people with the same or similar health condition and engaging in an enjoyable activity, such as singing, can reduce social isolation, and increase social support which in turn can reduce pain processing (Brown et al., 2003). We identified that singing interventions were well‐received and participants reported great positivity, which supports quantitative findings of reductions in pain interference (Figure 2). While the quantitative evidence of depression reduction was inconsistent, qualitative evidence highlights that singing greatly improved mood across all eight studies. The pathways by which singing improves pain processing appear to be non‐linear, and interwoven within those physical, psychological and social domains (Figure 3). Potential mechanisms of group singing should be further studied to gain a deeper understanding of how they are activated and complement each other; it would also be informative to examine how they relate to specific elements of group singing programmes (repertoire, being part of a group, facilitator, breathing, etc.) and result in greater benefits in pain patients.

Further, our syntheses suggest that the beneficial effects of group singing on depression appear to be disputable: only one RCT (Bradt et al., 2016) found a trend towards singing intervention, while other studies (Kenny & Faunce, 2004; Pongan et al., 2017; Tamplin et al., 2013) did not. This could be due to small sample size, high attrition rate, short intervention period and active comparator group. In contrast to our findings, a recent systematic review on singing for mental health indicated that singing reduced mental ill‐health with moderate to large effect sizes (Williams et al., 2018). A number of previous qualitative studies also affirm that participating in group singing can enhance mood and promote mental well‐being (Plumb & Stickley, 2017; Skingley, Martin, & Clift, 2015). Further, our qualitative synthesis suggests that patients have reported great psychological and social benefits from group singing. In our review, two qualitative studies (Bradt et al., 2016; Hopper et al., 2016) suggested that group singing interventions can reduce negative emotions and increase self‐efficacy. There is also evidence that suggests depression would be mediated by the relationship between social isolation and physical health, with higher social isolation scores predicting higher depression scores (Karayannis et al., 2018). As we presented in Figure 3, singing may have a knock‐on effect, such that reduced pain intensity, in turn, reduces pain interference and suffering, and consequently depression. In summary, there is sufficient qualitative evidence supporting singing intervention for reducing depression; inconsistent evidence from quantitative studies in this review warrants further investigation.

Moreover, our evidence syntheses suggest that studies with longer term interventions (>6 months) appear to be effective in reducing pain and depression, as interventions that lasted less than 6 months showed inconsistent findings. Thus, it can be recommended that patients with long‐term health conditions should take part in group singing for months and years. Additionally, future studies may assess high‐intensity (>1 hr weekly) short‐term intervention (<6 months), as there is currently no available evidence. Further, more studies are needed to establish appropriate singing intervention length and frequency for people with chronic health conditions in order to manage pain effectively.

The included studies have variable study quality with many having a number of weaknesses: in particular, internal validity, confounders and measuring adverse effects were identified as common deficiency. Included studies reported basic information, such as settings, facilitator's qualifications and repertoires (Table 1); however, more details of the protocol, the delivery of and compliance with their singing intervention were missing. It is important that researchers address internal validity in order to increase certainty that study findings can be attributed to the intervention (Horner, Rew, & Torres, 2006). Moreover, reporting these details assist future studies with designing their singing interventions, so that high‐standard singing interventions can be developed and delivered. Additionally, none of included studies addressed possible confounders, such as previous singing experience and expectation. Previous singing experience and expectations may have confounding impacts on the results, as people with previous positive singing experience and those who are aware of group singing benefits from popular media may have positive expectations, which in turn lead to positive outcomes. Another deficient quality issue of the included studies was measuring adverse effects. No study has reported their attempt to measure adverse events, but qualitative studies indicated that singing interventions were well‐received by patients.

### 4.1 Implications

The present review provides an analysis of the current evidence of the effects of singing on chronic pain management. More RCTs with active comparators with larger sample sizes are needed to better understand the potential effects of group singing on chronic pain for people with long‐term health conditions. Further, given the widespread popularity of singing and perception of potential health benefits, we advocate measuring participants' expectations prior to the singing intervention. Additionally, researchers need to identify a minimally required number of singing sessions, along with cost‐benefit analysis, so that group singing can be administered in the most effective way, to achieve optimal pain‐related outcomes for patients. Finally, future studies should have a fuller and more consistent approach to reporting the details of sessions, in order to identify potential mediators of the effects. For practice, this review affirms that group singing has a range of potential benefits for people with pain. Based on our synthesis, we recommend that at least 6 months of singing is most likely to be effective for reducing pain in people with long‐term health conditions. Group singing facilitators' roles are important, in order to engage participants fully into singing activities, as most people would require encouragement and support. Ongoing training and more resources are also required for group singing facilitators to provide the most effective singing sessions.

### 4.2 Limitations

Although all possible efforts were made to find relevant studies through major databases and other repository channels and having no language restrictions, the current review only included articles published in English, which may have presented a publication bias. The current review is also limited by the small number of RCTs and the inclusion of uncontrolled studies, which may produce overestimates of effects (Thornton & Lee, 2000).

## 5 CONCLUSION

Based on the research evidence syntheses presented in this review, currently there is limited support for the effects of group singing on chronic pain in people with long‐term health conditions. Group singing appears to have the potential to reduce pain intensity, pain interference and depression based on the limited corpus of studies with variable quality. Qualitative data in this review also highlighted that singing programmes were enthusiastically received by participants and had positive impacts on the physical, psychological and social aspects of participants' lives suggesting a variety of mechanisms. However, additional well‐designed studies are needed to investigate whether singing intervention has greater effects than other non‐pharmacological interventions on pain experience. Finally, given the wide‐ranging health benefits of group singing, practitioners are encouraged to continue this work and consider pain measures in evaluations of it.

## AUTHOR CONTRIBUTIONS

JYI: primary investigator involved in study selection, data extraction, analysis and manuscript writing.

DS: participated in searches, study selection, data extraction as a second reviewer, initial meta‐analysis and manuscript writing.

FB: participated in searches, study selection, quality assessments and manuscript writing.

DES: contributed to manuscript writing.

All authors discussed the results and commented on the manuscript.
