# Supplementary material for: A systematic review on the effects of group singing on persistent pain in people with long‐term health conditions
Source: Eur J Pain. 2019 Oct 15;24(1):71–90. doi: 10.1002/ejp.1485 (PMC6972717; doi:10.1002/ejp.1485)
Supplement: Supplementary file 1 [file EJP-24-71-s001.docx]

**APPENDIX 1. Search strategy for MEDLINE**

#1 (MH “Singing”) OR AB (“sing” OR “singing” OR “choir” OR “vocal exercise*” OR “singing group*” OR “karaoke” OR hymn OR “barbershop quartet”) OR TI (“sing” OR “singing” OR “choir” OR “vocal exercise*” OR “singing group*” OR “karaoke” OR hymn OR “barbershop quartet”)

#2 (MH “Chronic Pain”) OR (MH “Quality of life”) OR AB (“pain*” OR “quality of life” OR “psychological wellbeing” OR psychological well-being”) OR TI (“pain*” OR “quality of life” OR “psychological wellbeing” OR psychological well-being”)

#3 (MH “Chronic Disease+”) OR (“chronic condition*” OR “chronic disease*”)

#4 (MH ) OR AB (“cardiovascular disease*” OR “coronary heart disease*” OR “stroke” OR “cerebrovascular disease*” OR “hypertension” OR “hypotension” OR “peripheral vascular disease*” OR “embolism” OR “myocardial ischemia” OR “myocardial ischaemia”) OR TI (“cardiovascular disease*” OR “coronary heart disease*” OR “stroke” OR “cerebrovascular disease*” OR “hypertension” OR “hypotension” OR “peripheral vascular disease*” OR “embolism” OR “myocardial ischemia” OR “myocardial ischaemia”)

#5 (“cancer” OR “cancers” OR “neoplasm*” OR “melanoma” OR “leukemia” OR “leukaemia” or “lymphoma” OR “Hodgkin’s disease”)

#6 (“diabetes” OR “diabetes mellitus”)

#7 (“chronic respiratory disease*” OR “respiratory tract disease*” OR “COPD” OR “chronic obstructive pulmonary disease” OR “emphysema” OR “asthma” OR “chronic bronchitis” OR “chronic sinusitis” OR “apnoea” OR “bronchiectasis” OR “rhinitis”)

#8 (“musculoskeletal disease*” OR “arthritis” OR “osteoporosis” OR “osteopenia”)

#9 (“degenerative disease*” OR “osteoarthritis” OR “Alzheimer’s disease” OR “Parkinson’s disease” OR “Huntington’s disease” OR “multiple sclerosis” OR “amyotrophic lateral sclerosis” OR “dementia” OR “brain disease*”)

#10 (“inflammatory disease*” OR “rheumatoid arthritis”)

#11 (“chronic pain” OR “fibromyalgia”)

#12 (“chronic kidney disease*” OR “kidney disease*”)

#13 (“digestive system disease*” OR “cystic fibrosis”)

#14 #2 AND #3

#15 #2 AND #4

#16 #2 AND #5

#17 #2 AND #6

#18 #2 AND #7

#19 #2 AND #8

#20 #2 AND #9

#21 #2 AND #10

#22 #2 AND #11

#23 #2 AND #12

#24 #2 AND #13

#25 #14 OR #15 OR #16 OR #17 OR #18 OR #19 OR #20 OR #21 OR #22 OR #23 OR #24

#26 #1 AND #25
