# Supplementary material for: A systematic review on the effects of group singing on persistent pain in people with long‐term health conditions
Source: Eur J Pain. 2019 Oct 15;24(1):71–90. doi: 10.1002/ejp.1485 (PMC6972717; doi:10.1002/ejp.1485)
Supplement: Supplementary file 2 [file EJP-24-71-s002.docx]

**Appendix 2. Downs and Black Tool for Quality Assessment**

| **REPORTING** | **Yes/No/Partially** | **Score** |
| --- | --- | --- |
| 1. Is the hypothesis/aim/objective of the study clearly described? | Yes=1, No=0 |  |
| 2. Are the main outcomes to be measured clearly described in the Introduction or Methods section? | Yes=1, No=0 |  |
| 3. Are the characteristics of the participants included in the study clearly described? | Yes=1, No=0 |  |
| 4. Are the interventions of interest clearly described? | Yes=1, No=0 |  |
| 5. Are the distributions of principal confounders in each group of subjects to be compared clearly described? | Yes=2, Partially=1, No=0 |  |
| 6. Are the main findings of the study clearly described? | Yes=1, No=0 |  |
| 7. Does the study provide estimates of the random variability in the data for the main outcomes? | Yes=1, No=0 |  |
| 8. Have all important adverse events that may be a consequence of the intervention been reported? | Yes=1, No=0 |  |
| 9. Have the characteristics of patients lost to follow-up been described? | Yes=1, No=0 |  |
| 10. Have actual probability values been reported? | Yes=1, No=0 |  |
| **EXTERNAL VALIDITY** | **Yes/No/Unable to determine** | **Score** |
| 11. Were the subjects asked to participate in the study representative of the entire population from which they were recruited? | Yes=1/No=0/Unable to determine=0 |  |
| 12. Were those subjects who were prepared to participate representative of the entire population from which they were recruited? | Yes=1/No=0/Unable to determine=0 |  |
| 13. Were the staff, places, and facilities where the patients were treated, representative of the treatment the majority of patients received? | Yes=1/No=0/Unable to determine=0 |  |
| **INTERNAL VALIDITY - bias** | **Yes/No/Unable to determine** | **Score** |
| 14. Was an attempt made to blind study subjects to the intervention they have received? | Yes=1/No=0/Unable to determine=0 |  |
| 15. Was an attempt made to blind those measuring the main outcomes of the intervention? | Yes=1/No=0/Unable to determine=0 |  |
| 16. If any the results of the study were based on “data dredging, was this made clear? | Yes=1/No=0/Unable to determine=0 |  |
| 17. In trials and cohort studies, do the analyses adjust for different lengths of follow-up of patients, or in case-control studies, is the time period between the intervention and outcomes the same for cases and controls? | Yes=1/No=0/Unable to determine=0 |  |
| 18. Were the statistical tests used to assess the main outcomes appropriate? | Yes=1/No=0/Unable to determine=0 |  |
| 19. Was compliance with the intervention/s reliable? | Yes=1/No=0/Unable to determine=0 |  |
| 20. Were the main outcome measures used accurate (valid and reliable)? | Yes=1/No=0/Unable to determine=0 |  |
| **INTERNAL VALIDITY – Confounding (selection bias)** | **Yes/No/Unable to determine** | **Score** |
| 21. Were the patients in different intervention groups (trials and cohort studies) or were the cases and controls (case-control studies) recruited from the same population? | Yes=1/No=0/Unable to determine=0 |  |
| 22. Were study participants in different intervention groups (trials and cohort studies) or were the cases and controls (case-control studies) recruited over the same period of time? | Yes=1/No=0/Unable to determine=0 |  |
| 23. Were study participants randomised to intervention groups? | Yes=1/No=0/Unable to determine=0 |  |
| 24. Was the randomised intervention assignment concealed from both patients and health care staff until recruitment was complete and irrevocable? | Yes=1/No=0/Unable to determine=0 |  |
| 25. Was there adequate adjustment for confounding in the analyses from which the main findings were drawn? | Yes=1/No=0/Unable to determine=0 |  |
| 26. Were losses of patients to follow-up taken into account? | Yes=1/No=0/Unable to determine=0 |  |
| **POWER** | **Yes/No** | **Score** |
| 27^H^. Did the study carry out power calculation? | Yes=1/No=0 |  |
| **TOTAL SCORE** | |  |

^H^ = We modified this question from the original (“Did the study have sufficient power to detect a clinically important effect where the probability value for a difference being due to chance is less than 5%?”)
