# Supplementary material for: A systematic review on the effects of group singing on persistent pain in people with long‐term health conditions
Source: Eur J Pain. 2019 Oct 15;24(1):71–90. doi: 10.1002/ejp.1485 (PMC6972717; doi:10.1002/ejp.1485)
Supplement: Supplementary file 3 [file EJP-24-71-s003.docx]

**Appendix 3. Qualitative studies’ findings (k=8)**

| **1^st^ author (year)** | - **Data collection method (n= participant no.)** - **Data analysis method** | **Themes** | **Representative quotes** |
| --- | --- | --- | --- |
| Gale (2012) | - Interview (n=10) - Interpretative Phenomenological Analysis (IPA) | Friendship and support;  A common goal and focus for the future;  Improved mood, increased confidence, and self-esteem; *Sing for Life* is an extraordinary choir;  Physical changes;  Challenges and future directions | “I know my singing isn’t strong enough. I accept that, I’m happy with that. This choir is geared to allow everyone to come in.”  “You feel elated. It makes you feel uplifted. It makes you feel a lot better. And you forget that you’re there because you had cancer, really. It’s brilliant.”  “There’s lovely camaraderie there, from singing. It’s nice to have something all the family can do. It’s been good.” |
| Clements-Cortes (2013) | - Interview transcription + researchers field notes (n=28) - Phenomenological approach | Friendship and companionship; Simplicity;  Happiness, uplifting and positive feelings;  Relaxing and reduced anxiety; Fun | “I like to sing and sing with other people.”  “We sang songs that I like and know from the past.”  “I have never felt happier than when I am singing.”  “Singing in the choir was relaxing.” “Yes anxiety is reduced. After the first couple of sessions I started to enjoy it more and more.”  “I had so much fun each week singing with my new friends.” |
| Tamplin (2013) | - Interview (n=24) - Thematic analysis | Singing as a positive/pleasurable experience;  Singing increased confidence;  Singing in groups was motivating;  Singing was an independent accessible activity;  Changes occurred to their voice. | None was cited. |
| Clements-Cortes (2015) | - Interview (n=35) - Inductive analysis | Encouraged maximised participation;  Facilitating interaction and bonding;  Encourages improved mood and attitude;  Facilitates energy and motivation;  Promotes stress release and relaxation;  Recognised as therapy | “It was amazing to see my mother start singing these songs without even looking at the lyrics, she just knew them, I could see everyone getting something out of it.”  “Singing makes me feel happier”.  “For her, she has some pain, but sometimes it diverts her. It’s good. You know, music is really therapy”.  “There is no pain when I’m singing. When I’m feeling pain and I am singing. The pain stops”. |
| Bradt (2016) | - Focus group discussion (n=23) - Theoretical thematic analysis | Self-management; Togetherness; Transformation | “I never associated music with this part where I am dealing with pain all the time. I never thought that music could help pain. That was a great learning experience for me.”  “The self-care, the “me” time, I didn’t really have that in my life. So coming to visit the vocal class, I love it! It helped me how to deal with me.” |
| Fogg-Rogers (2016) | - Semi-structured Interviews (n=14) - A general inductive approach | Experience of their health condition;  Their subsequent needs; Experience of choir;  Perceived therapeutic benefits | “I enjoy the choir so much because it’s such a nice sort of positive, enjoyable thing to be able to do. That I can do.”  “Well, music suits everybody and language, communication, is such a vital part of life.”  “It’s lovely just to sit in there singing away too, and just to look around and watch everybody participating at their own level, in their own way, with no pressure.” |
| Hopper (2016) | - Semi-structured interviews (n= 7) - Thematic analysis | Physical improvement; Emotional impact;  Personal growth;  Interpersonal process; Relationship with the self; Living well with pain;  Sharing the music and spreading the word | “I don’t take my pills on a Friday because when I get there and start singing it sort of lifts you for the day”.  “When you’re singing you’re freer!”. “You come away and then for the rest of the day you’re sort of on a high”.  “The choir has helped me become that person again...my musicianship is now returning to me”. “It’s given me the confidence boost I needed to make decisions” |
| Reagon (2017) | - Interview (n=29); Focus group (n=20, 3 groups) - Thematic analysis | Singing is uplifting;  Positive distraction from cancer;  Increased ability to cope with cancer;  Improved self-worth, purpose and identity | “Singing must do something to your bold, to your endorphins or something, because it gives you a happy buzz.”  “The choir has given me a focus rather than sitting back and just feeling awful and feeling sad about things”. |
